# Distinct neural patterns for various information in working memory: A brain connectivity study

**DOI:** 10.1371/journal.pone.0326449

**Published:** 2025-07-03

**Authors:** Sadaf Sazesh, Ashkan Farrokhi, Vahid Shalchyan, Elizabeth L. Johnson, Mohammad Reza Daliri

**Affiliations:** 1 Neuroscience and Neuroengineering Research Lab., Biomedical Engineering Department, School of Electrical Engineering, Iran University of Science and Technology (IUST), Tehran, Narmak, Iran; 2 Cognitive Neurobiology Laboratory, School of Cognitive Sciences, Institute for Research in Fundamental Sciences, Tehran, Iran; 3 Departments of Medical Social Sciences, Pediatrics, and Psychology, Northwestern University, Chicago, Illinois, United States of America; Kochi University of Technology, JAPAN

## Abstract

Working memory (WM) relies on brain networks including the prefrontal cortex (PFC) and medial temporal lobe (MTL) as key nodes. Graph theory analysis has recently played an important role in uncovering brain connectivity architectures due to its ability to characterize complex brain networks. Yet, it remains unclear whether the PFC and MTL exhibit distinct effective connectivity patterns during information processing in WM. We employed graph theoretical analysis to investigate connectivity patterns involved in processing of various types of information (i.e., identity, spatial and temporal) in WM and predict behavioral reaction times (RT). Here, we hypothesized that WM processes identity, spatial, and temporal information via frequency-specific and regionally organized brain network mechanism. We analyzed intracranial EEG data from eight surgical epilepsy patients completing a WM task for everyday ‘what’, ‘where’, and ‘when’ information. To measure the effective connectivity between PFC and MTL, we used the directed transfer function and assessed the outputs for multiple graph theoretical metrics (i.e., degree, strength, clustering coefficient, eigenvector centrality, and betweenness centrality). Our findings reveal that theta-band oscillations predominantly support spatial and temporal information processing, with the PFC and orbitofrontal cortex (OFC) playing pivotal roles in spatial and temporal sequencing, respectively. The MTL was central to spatial and spatial-temporal integration. Alpha band connectivity was fundamental for spatial-temporal decoding, whereas beta and high-gamma bands were significant in RT differentiation, particularly in identity and spatial conditions. Notably, the PFC demonstrated widespread engagement across various graph metrics, underscoring its dominance in coordinating WM tasks and modulating cognitive processes. Our findings contribute to the broader understanding of WM’s neural mechanisms and offer insights into the dynamic coordination of brain regions supporting cognitive tasks.

## Introduction

Working memory (WM) refers to the capacity to temporarily hold and process information [1]. The prefrontal cortex (PFC) is known for coordinating of cognitive control and support a variety of higher cognitive functions [2]. Within the PFC, single neurons maintain representations of task-relevant stimuli in WM [3,4]. Analysis of a population of dorsolateral prefrontal cortex neurons has investigated dynamic activity patterns during the delay period of a delay-response task, highlighting their importance in information maintenance and manipulation essential for WM [5–7]. Lesions in the PFC result in profound deficits across a wide range of functions, including WM, learning, planning, attention, and motivation [4,6,8–12].

Numerous functional magnetic resonance imaging (fMRI) studies have revealed the PFC’s role in maintaining various types of information within WM. Sustained PFC activity has been observed during the maintenance of spatial information, and distinct PFC activation patterns have been linked to spatial and nonspatial information maintenance [13–16]. Notably, studies on monkeys have shown that PFC neurons encode not only the content of information but also its duration and even irrelevant content in WM processes [17]. These findings demonstrate that the PFC’s role in WM extends beyond simple information storage, encompassing complex processes such as encoding, maintenance, and manipulation of diverse stimulus information [4,18].

WM depends on a network of brain regions, including the PFC and the medial temporal lobe [19]. The hippocampus and surrounding medial temporal lobe (MTL) structures are responsible for coding and retrieving our events, which is known as episodic memory. Recent research suggests that the MTL contributes to WM, especially in spatial information [20].

Neurophysiological studies in both humans and animals have revealed the crucial role of the MTL in everyday experiences by connecting with specific brain regions in both the cortex and subcortical structures [21] [22] [23]. In particular, research on rats has shown that they utilize a combination of spatial (“where”) and olfactory (“what”) cues to recognize when events occur. With lesions in the hippocampus disrupted the integration of “what,” “where,” and “when” information within the memory tasks [22].

A study in line with our dataset and task indicated that there is a specific mechanism in the MTL responsible for processing space and time, as well as a more general mechanism in the PFC in the theta band. These mechanisms work together through rapid and dynamic interactions to support WM for everyday experiences [24]. Another study showed that the analysis of spatiotemporal information revealed heightened connectivity in the delta-theta range compared to identity information processing. Conversely, alpha-beta connectivity remained insensitive towards WM contents [25]. These findings inspire further investigation of neuronal networks within frequency bands in WM.

Moreover, the neuropsychological findings emphasize the role of the orbitofrontal cortex (OFC) in executive control functions which is the storage and recall of temporal information within long-term memory [26–28]. Studies involving OFC lesions in line with the same task revealed the impairment in temporal sequence memory without impairments in spatial WM or overall cognitive performance [29]. Another paper related to this task investigated the role of the brain in prioritizing spatial and temporal information in WM. Researchers found that collaborations of alpha and gamma activity in parieto-occipital regions were involved in selecting specific information, While accuracy in subjects with PFC lesions was impaired [14,30].

In this study, we aimed to investigate how distinct (WM) processes; spatial, identity, and temporal are supported by specific patterns of brain network connectivity across different frequency bands and graph metrics. Specifically, we formulated the following hypotheses: decoding performance (classification accuracy) would differ across frequency bands, with certain frequency bands (e.g., theta and alpha) showing higher decoding accuracies for specific WM task types (spatial, identity, temporal). Among those frequency bands with statistically significant decoding accuracies (p < 0.05 compared to the average across all bands), graph-theoretical metrics (e.g., degree, strength, eigenvector centrality) would explain and differentiate the network mechanisms underlying each type of WM process. Spatial processing would predominantly engage theta band connectivity within visuospatial and PFC regions, supporting attentional and maintenance processes. Identity processing would involve beta band activity and increased PFC and OFC network metrics, reflecting feature-binding and identity maintenance. Temporal processing would rely on theta and alpha connectivity, with the MTL and OFC contributing to sequence encoding and temporal control. These hypotheses allowed us to first decode WM processes based on brain connectivity, and then clarify the contribution of specific frequency bands and network graph metrics to each cognitive operation.

Graph theory metrics complete these oscillatory patterns by capturing the global and regional connectivity required for WM processes. We assessed central brain regions and level of local connectivity in WM networks by degree and clustering coefficient, respectively. In addition, we investigated the influence of brain regions in transferring information by the Strength. We specified network hubs that play a central role in information processing and a bridge for information flow among nodes by eigenvector and betweenness centrality, respectively.

Intracranial electroencephalography (iEEG) including subdural and depth recordings from surgical epilepsy patients, provides a unique opportunity to investigate human cognition with high temporal and spatial resolution [20,31,32]. These recordings demonstrated that brain networks are included in distinct aspects of WM processing [19]. In this study, we analyzed an online published iEEG dataset. The dataset contains recordings from PFC, MTL, and OFC during performance of a visuospatial WM task for everyday ‘what’, ‘where’, and ‘when’ information [24]. Effective connectivity patterns between PFC and MTL during WM processing can be analyzed using the directed transform function (DTF). DTF allows us to investigate frequency-specific connectivity patterns underlying information processing within these networks [33–36]. The primary objective of our study is to analyze brain connectivity using graph theory in a WM task and investigate how different brain regions communicate and cooperate during cognitive processes. Then we aimed to specify neural correlates of WM performance as reflected in RT [37]. By using graph metrics across five frequency bands and DTF analysis, we aim to perform comparisons (identity vs. spatial, identity vs. temporal, spatial vs. temporal). Through these analyses, we identify neural patterns specific to each type of processing, while also studying the relationship between these information types and subjects’ RT. Through feature selection and the classification of fast and slow RT, we aim to illustrate the importance of various information types in predicting subjects’ behavior.

Our results demonstrate the prominent role of theta band, then alpha and beta in processing everyday information we experience. Furthermore, our analysis explored the distinct patterns in the MTL and frontal regions relation to WM-related information and behavioral outcomes. These findings align with our theoretical predictions for distinct patterns in the MTL and PFC regions relation to WM-related information in various frequency bands, then study the complex interplay between WM processes and behavioral outcomes.

## Materials and methods

### Subjects

**Fig 1:**
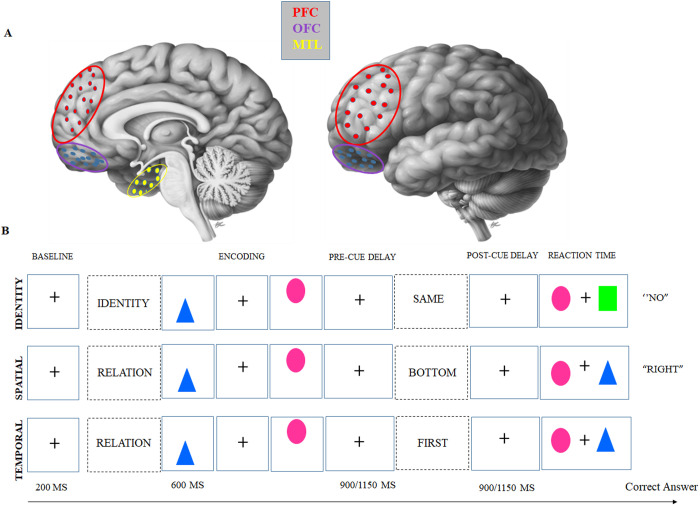
The structure of 1 task with 3 trial types in random order and regions. **(A)** iEEG coverage of three regions of interest from the lateral and medial views of brain. (B) Visuospatial task, During the encoding phase, subjects were shown two shapes one after the other (each for 200 ms, with a 200-ms gap in between) in an arrangement where one shape was above the other. Following a maintenance interval that lasted between 900 and 1150 ms, subjects were prompted to recall either the shape that was displayed in the top or bottom position (spatial), or the shape that was shown first or second (temporal). This occurred during a post-Cue processing interval of the same duration. In a third of the trials, subjects were instructed to keep a mental image of the shapes (identity).

The dataset analyzed in this study includes iEEG recordings obtained from the CRCNS (http://dx.doi.org/10.6080/K0VX0DQD) database, recorded by Elizabeth Johnson and her colleagues [24]. The dataset contains recordings in 10 adults (mean ± SD [range]: 37 ± 13 [22–69] y; 7 males) being monitored with iEEG. Each subject performed a visuospatial WM task with channels in the MTL (i.e., CA1; CA3/dentate gyrus; subiculum; or parahippocampal, perirhinal, or entorhinal cortex), lateral PFC (inferior, middle, or superior frontal gyrus), and OFC (orbitofrontal, frontal polar, or medial prefrontal cortex). The dataset comprised of 254 virtual electrodes, with an average (± standard deviation) per subject: 4 (± 2) in MTL, 12 (± 8) in PFC in 9 out of 10 subjects, and 10 (± 14) in OFC in 9 out of 10 sujects. For the purpose of this study, we included data from 8 out of 10 subjects to ensure the inclusion of recordings from all three regions of interest (Fig 1A).

### Behavioral task

Subjects performed a WM task including spatiotemporal relation and identity trials. On spatiotemporal relation trials, subjects maintained information about which shape had been previously indicated in the top/bottom spatial or first/second temporal order. In identity trials, subjects maintain a representation of the identity of both shapes regardless of spatiotemporal order [10,24,25,38]. The trials randomly intermixed in a single test session. Following a 1-second fixation interval, a starting screen appeared for 800 ms to indicate whether the upcoming stimuli would be tested for “identity” or spatiotemporal “relation”. Then, there was a central fixation interval of 100 ms before presenting two common-shape stimuli in specific spatiotemporal configurations (top/bottom spatial and first/second temporal positions) for 200 ms each. After either a 900- or 1,150-ms delay interval (randomly jittered), the test cue was presented for 800 ms to elicit information-specific selection mechanisms during another delay interval of the same length. This random jittering ensured that anticipatory mechanisms were precluded. Finally, subjects responded to a two-alternative forced choice test with two shapes presented on the horizontal axis, resulting in an equal chance rate of choosing correctly. During the identity trial, subjects were asked to determine if a pair of shapes was the same as the pair they had just studied (“Yes” or “No”). Half of the pairs contained of two old shapes, while the other half consisted of one old shape and one new shape. Subjects indicated their answers by using either the up or down arrow keys. For spatial relation trials, subjects used left and right arrow keys to indicate which shape was on “top” or “bottom”. In temporal relation trials, subjects used left and right arrow keys to specify which shape was presented “first” or “second” (Fig 1B). There were 120 trials, where the identity trials are considered as the identity condition, the spatial trials as the spatial condition, and the temporal trials as the temporal condition. Task was completely balanced with an equal distribution of 120 trials between the identity, spatial, and temporal trials (mean ± SD [range] each condition: 34 ± 3 identity trials, 35 ± 3 spatial trials, 33 ± 3 temporal trials). Subjects demonstrated high proficiency in the task, with accuracy ranging from 0.79 to 0.97 (chance: 0.5). This proficiency provided a strong basis for subsequent analysis which examines neural correlates of performance and relationships between behavior and neural activity [24].

### Data analysis

Only correct trials were included in the further analyses. Since certain subjects had more electrodes in specific areas than others. Connectivity metrics were calculated for MTL-PFC, MTL-OFC, and PFC-OFC electrode pairs. Then, graph metrics were measured for each subject’s data. To address the variability in electrode coverage across subjects (4 PFC + 4 OFC + 1 MTL channels vs. 1 PFC + 1 OFC + 4 MTL channels), which could influence graph theoretical metrics, we averaged graph values within each brain region. This resulted in 27 values per subject (3 regions * 9 graph metrics). The analysis was conducted based on these averaged values, and a 600 ms window in the post-cue delay (300–900 ms after the test cue appeared) was selected to avoid stimulus-evoked responses [39]. Connectivity matrix was then measured for each trial, and a classification analyses were performed to differentiate between conditions among subjects.

### Effective connectivity analysis

DTF was used to investigate the directional influence between brain regions, enhancing our understanding of the complex connectivity patterns within the brain [40]. We used iEEG signals of eight out of ten subjects as the input of DTF and the output of DTF was the connectivity matrix. The following section describes a brief theoretical basis for the DTF method.

For a multivariate k-channel process *X(t)*:


$$\begin{array}{c} \;X\left(t\right)={\left(x\left(t\right),x_{2}\left(t\right),\cdots ,x_{\text{k}}\left(t\right)\right)}^{T} \end{array}$$ (1)

The multivariate autoregressive (MVAR) model on this process takes the form:


$$X\;(t)=\;\sum_{m=1}^{p}\hat{A}(m)\cdot X(t-m)+E(t)\;or\;\sum_{m=0}^{p}A(m)\cdot X(t-m)=E(t)$$ (2)

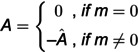

(3)


Where E(t) is a vector of multivariate zero-mean uncorrelated white noise process, A(mis the k×k matrix of the model coefficients and the P is the model order. To investigate the spectral relation of the process, the equation can be easily transformed to frequency domain:


A(f)X(F)=E(f) (4)

$$X(f)=A^{-1}(f)E(f)=H(f)E(f)\;$$ (5)

$$E(f)=\sum_{m=0}^{p}A(m)\cdot {exp\;}^{-i2\pi \cdot f\cdot m}$$ (6)

From the equations, the process can be considered as a linear filter with white noise as its input. Therefore, the matrix of coefficients is called the transfer matrix. Elements of transfer matrix contain information about all relation between signals of data channel where represent the connection between *j*^th^ input and *i*^th^ output of the system.

The causal influence of channels could be measured by DTF, which is a normalized version of the transfer function and defined as:


$${DTF}_{j\to i}^{2}(f)=\frac{{\left|H_{ij}(f)\right|}^{2}}{\sum_{m=1}^{K}{\left|H_{im}(f)\right|}^{2}}\;$$ (7)

Where $H_{ij}$ represents the element of the ith row and jth column of matrix **H**. We fitted a MVAR model on each trial of data using ARFIT toolbox. As order selection criteria, the ARFIT computes an approximation to Schwarz’s Bayesian criterion (SBC) [41]. Then the connectivity matrices calculated for theta (4–8 Hz), alpha (8–12), beta (12–30 Hz), low-gamma (30–80 Hz) and high-gamma (80–200) [42] as:


$$C_{frequency\;band}=mean\left({DTF}^{2}(f)\right)$$ (8)

f ∈ frequency band

### Graph theoretical analysis

Graph theory serves as a valuable tool for modeling brain networks [43]. The human brain is widely conceptualized as a graph, consisting of nodes and their connections as edges, a multitude of elements that dynamically interact to facilitate the transmission of information [44]. This framework has been widely applied in neuroscience to investigate the neural patterns of cognitive processes, including WM [45].

By treating connectivity data as graph-theoretical networks, it permits analysis of groups of three or more units and better captures the real-world system of the brain than do binary interactions between channel pairs [46]. In this context, a simplified representation of the brain can be achieved by defining a graph as a collection of nodes (here, iEEG channels) and internode connections or edges (DTF) [44,47]. Each element of connectivity matrix, DTFij, represents the effective (direct) influence from node j to node i.

The selected graph metrics included degree, in-degree (input information), out-degree (outflow of information), strength, in-strength (weights of incoming edges), out-strength (weights of outgoing edges), clustering coefficient, betweenness centrality, and eigenvector centrality. For clarity, in-degree, out-degree, in-strength, and out-strength are treated as independent metrics, contributing to the total number of metrics analyzed. These graph metrics were computed using the Brain Connectivity Toolbox (BCT) [21], a widely-used software package specifically designed for analyzing brain connectivity data [48]. To calculate the graph theory metrics, it is necessary to utilize thresholded connectivity matrices. This step involves selecting a threshold value to determine the significance of connections between nodes. It exhibits substantial functional interactions while excluding weaker links. Additionally, maintaining a consistent density of connections across all trials is crucial for conducting meaningful comparisons. This involves matching the number of connections or edges in the graph across different trials. By doing so, we ensure that the comparisons are not biased by variations in network density [49,50].

For each frequency band, a distinct threshold was applied to account for the variations in average connectivity values across different frequency bands [51]. This approach ensures that the thresholding process is tailored to the characteristics of each frequency band. To apply the thresholding, the effective connectivity metrics were adjusted by preserving a certain percentage of the strongest connections, while setting the remaining connections to zero. This proportional thresholding (PTh) method involves determining the ratio between the preserved edges and the total number of edges in the network [52].

To identify the most important connections among three types of conditions, a range of PTh values from 0.01 to 0.5 (with a step size of 0.01) was initially examined on connectivity matrices [52–54]. This allowed for an exploration of the various threshold levels and facilitated the identification of connections that exhibited the highest effect [49]. Finally, we selected the range of 0.06–0.2 for PTH for each band and graph metrics; owning the impact of thresholding on graph metrics. Notably, thresholds were selected according to the highest decoding accuracy from the mentioned range in each condition and then further analysis was done according to selected threshold [55].

### Degree

The degree of node i (d_i_) in a directed network includes out-degree and in-degree which is described as the number of edges a node has with the other nodes [56]. The out-degree of node i (od_i_) is an outflow of information from node *i* to other connected nodes. The in-degree of node i (id_i_) is quantified as the input information from the rest of the networks to node i [40,57] formally,


$${id}_{i}=\;\sum_{j\neq i}W_{ji}$$ (9)

$${od}_{i}=\sum_{j\neq i}W_{ij}$$ (10)

and


$$d_{i}={id}_{i}\;+{od}_{i}$$ (11)

W can be defined as the information or weights in a network. These weights can represent the connections between different nodes in the network.

### Strength

The strength of a node is calculated by summing the weights of all edges connected to that particular node. To focus on the strong connections, we introduced a modified metric called ‘weighted strength.’ This metric is calculated by summing the weights of edges above the proportional threshold, and it takes into account both the in-strength and the out-strength of the node. The in-strength of a node is defined as the sum of the weights of incoming edges, indicating the total amount of information received by the node from other nodes in the network. The out-strength of a node represents the sum of the weights of outgoing edges, reflecting the total amount of information above PTH which is transmitted from the node to other connected nodes [56,58]. Strength of a node is a sum of the out-strength and in-strength.

By considering both the in-strength and out-strength, the node strength metric provides a comprehensive assessment of the node’s influence within the network. It enables the identification of nodes that play a higher role in the flow of information, as nodes with higher strength values have a greater impact on the overall connectivity and dynamics of the network.

### Clustering coefficient (CC)

The clustering coefficient serves to capture the local connectivity patterns within a network, particularly the tendency for nodes’ neighbors to form clusters or triangles, while degree and strength provide information about how well-connected individual nodes are in a network. It helps uncover important aspects of network structure and behavior, making it a useful tool in graph analysis [59]. The CC of node *i* is the ratio of present connections between edges that are connected to node *i* to the number of all possible connections between these connected nodes [60,61]:


$${clustcoeff}_{i}=\frac{2\sum_{j=1}^{n}v_{ij}}{n_{i}(n_{i}-1)}$$ (11)

In which, a value of 1 is assigned to $v_{ij}$ if there is a connection between *i* and *j* and 0 otherwise, and *n* is the number of nodes that are connected to node *i*.

### Betweenness centrality (BC)

betweenness centrality in brain network analysis provides insights into the influence of specific brain regions in terms of their role as communication hubs.

The BC of a node in a network is considered as the ratio of the shortest path that passes through the node v to the shortest path in the network [57]:


$$BC(v)=\sum_{i\neq j\neq v}\frac{\sigma_{ij}(v)}{\sigma_{ij}}$$ (12)

$\sigma_{ij}$ refers to the number of all shortest paths between node *i* and *j*; $\sigma_{ij}(v)$ is the number of the shortest path that passes through the node *v*. Nodes with high betweenness centrality act as hubs in the network. In contrast, BC focuses on the role of a node in facilitating communication and information flow along shortest paths within the network. Eigenvector centrality identifies nodes that act as critical bridges or intermediaries, which is described as follows [62].

### Eigenvector centrality

Eigenvector centrality measures the influence of a node in a network by considering not only the number of connections it has but also the centrality of its neighbors. The sum of neighbors’ centralities of the node *i* is defined as the eigenvector centrality [63]. To calculate, if *A* is considered as an adjacency matrix, C_E_(*i*) is an eigenvector that with multiplication by *A*, the equation of Ax=λx is satisfied [64]:


$$C_{E}(i)=\;x_{i}=\frac{1}{\lambda_{1}}\sum_{j=1}^{N}A_{ij}x_{j}$$ (13)

The scalar *λ* is an eigenvalue of *A* which $C_{E}(i)$ of node i is measured as the ith input in the eigenvector matrix that belongs to the largest eigenvalue of A that is marked by $\lambda_{1}$.

### Supervised machine learning

The application of machine learning methods for examining brain networks, especially in the context of WM tasks, offers several benefits. These methods enabled the identification of optimal feature combinations and the prediction of behavioral outcomes. In this study, we applied machine learning models to select the most discriminative graph metrics and brain regions associated with different WM processes, thereby focusing on the key neural correlates of WM through feature selection techniques [65].

For further analysis, a supervised machine learning algorithm was employed. All values were normalized between 0 and 1 with Z-score normalization [66]. Within each region (MTL, PFC, and OFC), the graph metrics (degree, in-degree, out-degree, strength, in-strength, out-strength, clustering coefficient, betweenness centrality, and eigenvector centrality) were measured, and as a result, there were 27 values for training using post-cue delay. Due to frequency-specific effects, these values were extracted for each frequency band and conditions were then classified using different thresholds.

To ensure an adequate number of values and a balanced representation of different classes in our classification task, we employed the relief algorithm. This iterative feature selection method considers interactions between features and assigns importance scores based on differences in feature values between neighboring samples [67]. The relief algorithm calculated feature scores, ranked them, and determine the optimal number of values that effectively discriminated between the two conditions. This process helped identify the most relevant features contributing to classification performance. Additionally, there exists a tradeoff between the number of trials and values. These selected values were then normalized between 0 and 1 to ensure a consistent scale across all values.

We used a random subsampling approach for the validation of our classification process. This involved repeating the process 100 times, where each repetition randomly selected 80% of the trials as the training set and the remaining 20% as the test set. Values were normalized based on the training set, ensuring consistent scaling across both sets [68]. During each iteration, feature selection was performed on the training set to identify the most informative values for classification [69]. These selected values were then used to train the classification model, which was subsequently tested on the corresponding test set. We investigated different classifiers in MATLAB to identify the best classification algorithm which has high predictive accuracy; therefore, a support vector machine (SVM) was chosen with a linear kernel for further analysis. Briefly, having training examples, SVM forms a hyperplane as a decision-making surface so that the margin of separation between positive and negative samples become maximized, and values become linearly separated [70].

To assess the statistical significance of our classification results, we employed a permutation test. This involved creating 1000 shuffled datasets by randomly shuffling the data labels. For each shuffled dataset, the entire classification analysis, including feature selection and training with the shuffled labels, was repeated. To compare the accuracies obtained from the correct labels with the accuracies from the shuffled labels, we employed the Wilcoxon Rank-Sum test. This test determined whether the observed accuracies were significantly higher than what would be expected by random chance alone [71]. This comprehensive validation process allowed us to evaluate the performance and generalization of our classification model. By repeating the analysis multiple times with different train-test splits and comparing the results to shuffled datasets, we obtained reliable estimates of classification accuracy and assessed the significance of our findings.

In our analysis, the Kruskal-Walli’s method was used to compare decoding accuracies of eight subjects between bands. For post-hoc pairwise comparisons, we used MATLAB’s multcompare function and manually specified the Dunn-Sidak correction to account for multiple comparisons in our nonparametric data. These tests provide a robust analysis to determine if there are significant variations in decoding accuracies among the subjects within each specific frequency band, especially in clinical data, because they provide a nonparametric framework for detecting significant differences in decoding accuracies across subjects and frequency bands [72]. The Kruskal-Wallis test is an extension of the Wilcoxon Rank-Sum test from two samples to multiple samples [30].

After each iteration, each feature was assigned a value of zero if it was not selected and one if it was selected through the feature selection process. This procedure was repeated 100 times. By summing these values across the iterations, each of the 27 values obtained a cumulative score ranging from zero to one hundred. This scoring algorithm was applied to each individual subject and to the average across subjects. Subsequently, the frequency band with the highest decoding accuracy was identified.

### Reaction time analysis

**Fig 2:**
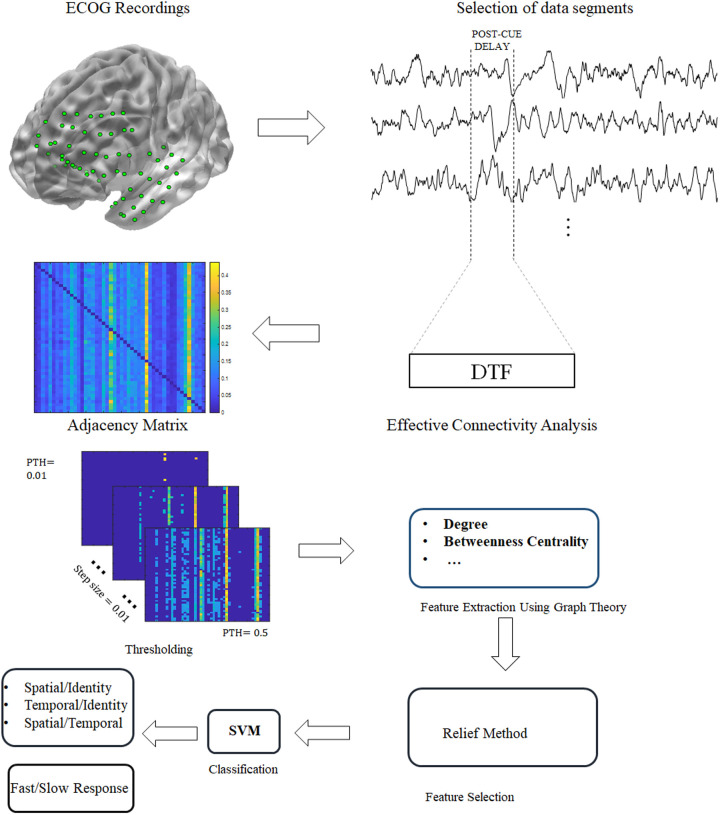
Proposed scheme for utilizing classification approaches. Initially, ECOG recordings were extracted from subject number 6. We then used the POST-CUE delay for our study analysis and applied a DTF functional connectivity analysis on it. After extracting the adjacency matrix, we needed to apply thresholding (PTH) from the range of 0.01 to 0.05. Feature extraction was applied based on graph theory and then Relief feature selection was performed. The classification of conditions and fast and slow RT was done by the SVM classifier.

The experiments conducted in this study included three different conditions: identity, spatial, and temporal. Each subject exhibited varying RT in these conditions and every trials. To facilitate the classification process and ensure an adequate number of trials, the RT were categorized into two categories: slow and fast. Since some subjects naturally respond faster than others for reasons unrelated to task performance (e.g., clinical procedures affecting a patient’s use of their dominant hand), it was necessary to control for inter-subject variability, we normalized data per subject and then combined RT from all subjects within each condition (e.g., identity condition). The combined RT were then sorted in ascending order, and the first quarter and the last quarter of the values were assigned to the fast and slow classes, respectively [73]. The number of trials categorized as fast and slow was 68 trials each for the identity condition (136 total trials), 142 trials for spatial and 130 trials for temporal conditions. Subsequently, all the aforementioned analyses for feature extraction and feature selection were applied to classify the fast and slow classes. This involved computing graph-based measures, performing feature selection, and training a classification model to capture optimal feature combinations and the prediction of behavioral outcomes (Fig 2).

### Statistical analysis

To investigate the differences across inter-regional and graph metric among eight subjects, a two-way repeated-measures ANOVA was conducted with two within-subject factors: Regions and Graph Metrics. This analysis was applied to the frequency of use of all 27 features across the eight subjects. Matching was performed across both factors to account for repeated measurements within subjects. When the assumption of sphericity was violated, the Greenhouse-Geisser correction was applied.

The significance level was set at α = 0.05 for all statistical tests. Pairwise comparisons were performed using Tukey’s multiple comparisons test to evaluate differences between all groups. This test was chosen because it adjusts for multiple comparisons, controls the Type I error rate, and is appropriate for balanced designs. Adjusted p-values were reported to account for the family-wise error rate. For balanced designs. Adjusted p-values were reported to account for family-wise error. All statistical analyses were performed using Prism 10.0 and MATLAB. Data are presented as mean ± standard error of the mean (SEM).

To assess the significance of individual features in classifying fast and slow trials, we analyzed the frequency of feature selection. In each iteration of the classification process, a feature selection algorithm identified 9 out of 27 features. This process was repeated 100 times. For each iteration, a binary vector indicated whether each feature was selected or not. To determine whether individual features were selected more frequently than expected by chance, we calculated a statistical threshold based on the binomial distribution. The null hypothesis assumed that each feature had an equal probability of being selected, p = 1/3, at each iteration. The cumulative binomial probability was used to compute the critical value for a significance level of α = 0.05, representing the minimum number of selections required for a feature to be considered significantly more frequent than chance. Specifically, the critical value was determined as the smallest k such that:


P(X≥k)≤α or equivalently P(X<k)≥1−α (14)

where k is the number of times a given feature is selected over 100 iterations, n = 100 is the total number of iterations, and p = 1/3. The formula for the cumulative probability of the binomial distribution is given by:


P(X<k)=∑x=0k−1(nx)px(1−p)n−x (15)

The resulting critical value was determined to be 43. Any feature selected 43 or more times out of 100 iterations was considered significantly more frequent than expected by chance. Features selected fewer than 43 times were not considered to exceed chance levels. The analysis revealed features that were consistently important for distinguishing between fast and slow trials, offering insights into their relevance for the classification task.

## Results

### Differences between eigenvector centrality and strength in PFC during spatial and identity processing, particularly within the theta band

**Fig 3:**
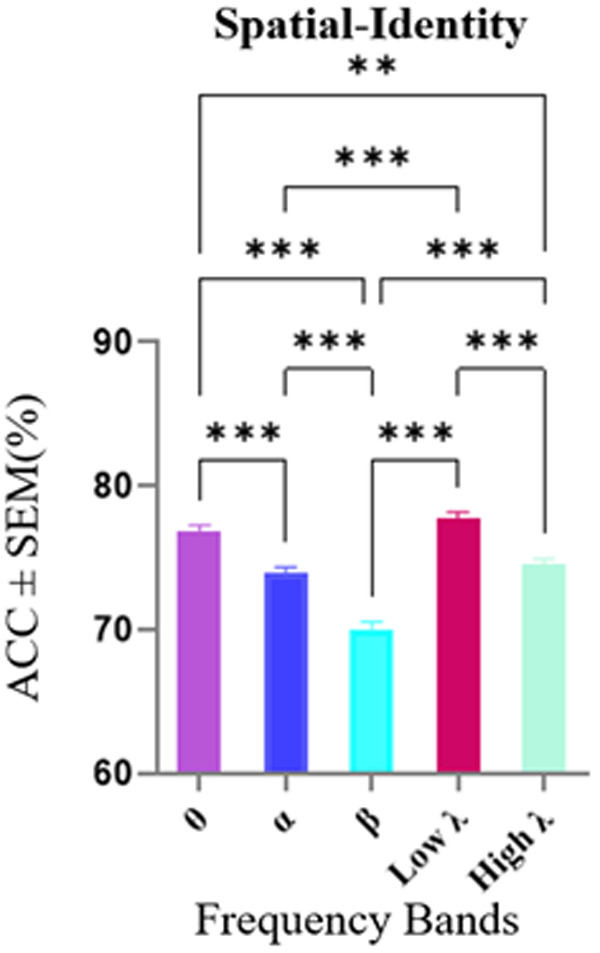
Decoding accuracy in Spatial-Identity condition across frequency bands. The horizontal axis shows different frequency bands and the vertical axis shows the average accuracies ± SEM. The average decoding accuracies in the low-gamma, and theta frequency bands were significantly higher than other bands. Data were analyzed by Kruskal-Wallis test and multiple comparison tests (**) p < 0.01, (***) p < 0.001, (****) p < 0.0001.

Decoding of spatial and identity conditions was initially assessed separately for each subject, due to the variation in the number of electrodes across subjects. Subsequently, we averaged across subjects for a comprehensive view and insights into the overall trends and variabilities observed in the different frequency bands. Mean ± Standard error of the mean (SEM) values of decoding are presented in frequency bands, as depicted in Fig 3. The results indicate that in the Spatial-Identity conditions, decoding accuracy differed significantly across frequency bands (Kruskal-Wallis test, p < 0.001). Post-hoc pairwise comparisons using MATLAB’s multcompare function with Dunn-Sidak correction revealed that decoding accuracy in the low-gamma band (77.72%) was significantly higher than in the alpha, beta, and high-gamma bands (all p < 0.001). Additionally, the theta band (76.85%) showed significantly higher decoding accuracy than the alpha and beta bands (p < 0.001), and also higher than the high-gamma band (p < 0.01).

Based on the results shown in Fig 3, which represent the mean accuracy across frequency bands for eight subjects, we computed the mean decoding accuracy across all bands and statistically compared each band’s accuracy against this mean using a Wilcoxon signed-rank test. The results showed that both theta and low-gamma bands demonstrated decoding performances significantly above the mean (p < 0.05). However, in subsequent feature selection method, only the theta band produced statistically meaningful and consistent effects, while analyses in the low-gamma band did not yield significant results. Therefore, we focused our subsequent analyses on the theta band. The results for low-gamma, is provided in the supplementary (S1 Fig) for completeness.

**Fig 4:**
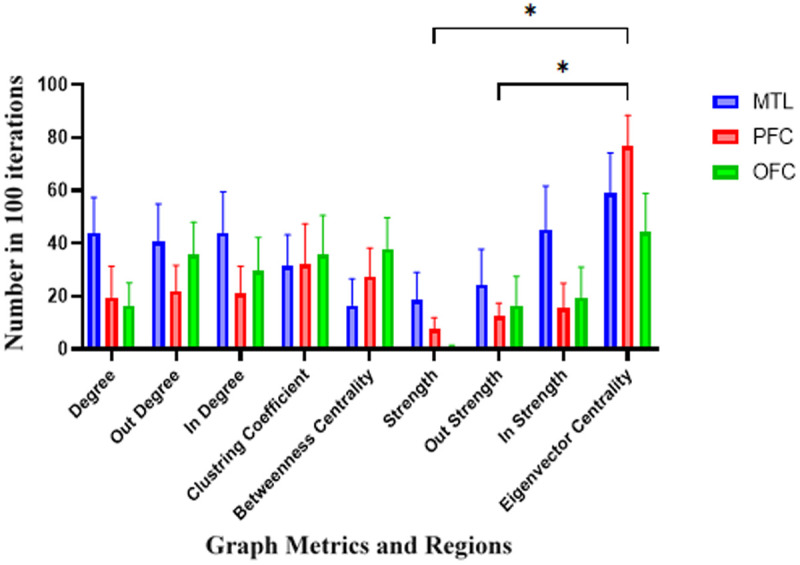
Feature importance in Spatial-Identity conditions in the theta band. Each feature was assigned a score ranging from 0 to 100, reflecting its importance in the classification process. The horizontal axis shows different graph metrics and the vertical axis shows the mean of scores among 100 iterations. Results of a two-way repeated measures ANOVA on theta band decoding data with region (MTL, PFC, OFC) and graph metric as factors. Significant interactions were observed in graph metrics within the PFC, with differences between strength and eigenvector centrality (p = 0.02, Mean Difference = −69.1) and out-strength and eigenvector centrality (p = 0.02, Mean Difference = −64.5). No significant differences were detected in other regions or graph metrics in the low-gamma band. Abbreviations: MTL, medial temporal lobe; PFC, prefrontal cortex; OFC, orbitofrontal cortex.

In the feature selection method that conducted over 100 iterations, each features took a 0–100 score. A two-way repeated measures ANOVA was conducted within region (MTL, PFC, OFC) and graph metric as factors to analyze the data of Spatial-Identity conditions. Post-hoc Tukey’s multiple comparisons tests revealed the following significant results: associate to Fig 4, in the theta band, we observed a significant interaction in graph metrics with p = 0.035, F (2.89,20.2) =3.52. Specifically, in the PFC, differences were noted for strength versus eigenvector centrality (p = 0.02, Mean Difference = −69.1) and in out-strength versus eigenvector centrality (p = 0.02, Mean Difference = −64.5). No significant differences were detected among other regions. In the lower gamma band, no differences were observed between regions, graph metrics, or interactions. For the plot showing mean of scores in each graph, the horizontal axis represents different graph metrics, and the vertical axis shows the number of “1” value in 100 iterations for each metric across the regions.

These findings align with our first hypothesis, which predicted that decoding performance would differ across frequency bands, with the theta band showing enhanced decoding for spatial-identity conditions. The observed significant differences between strength and eigenvector centrality in the PFC within the theta band further support our third hypothesis, which posited that spatial processing would predominantly engage theta band connectivity within visuospatial and PFC regions, reflecting attentional and maintenance mechanisms. These results highlight the pivotal role of the PFC’s theta band connectivity in supporting spatial-identity processing in working memory.

### Theta band contributions and regional differences in graph metrics support temporal and identity processing

**Fig 5:**
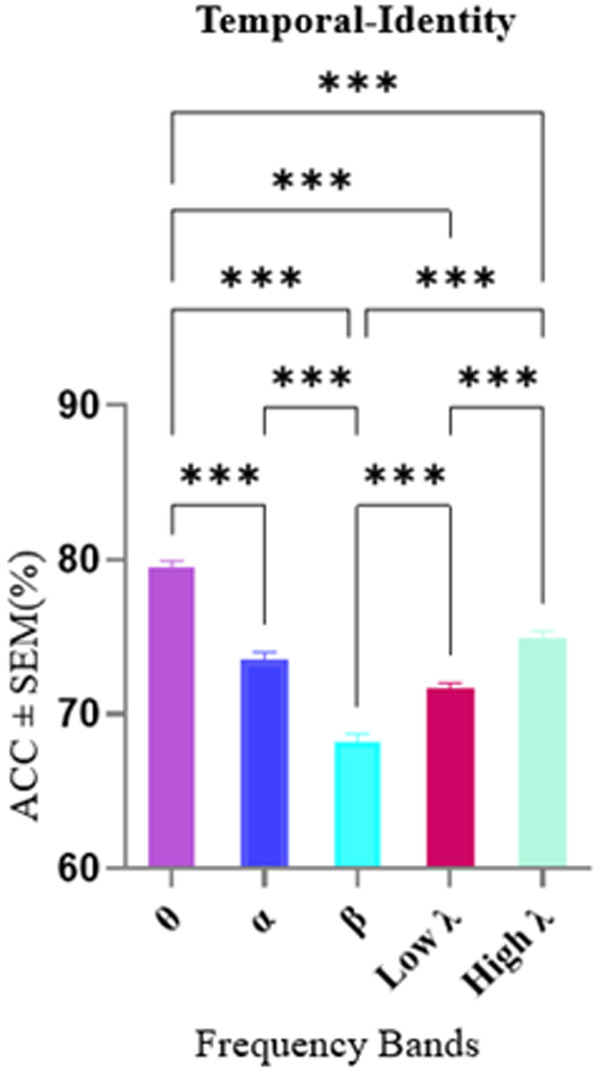
Decoding accuracy in Temporal-Identity condition across frequency bands. The horizontal axis shows different frequency bands and the vertical axis shows the average accuracies ± SEM. The average decoding accuracies in theta frequency band were significantly higher than other bands. Data were analyzed by Kruskal-Wallis test and multiple comparison tests (*p) <0.05, (***) p < 0.001, (****) p < 0.0001.

To differentiate between temporal and identity conditions, we employed the same methodologies. Fig 5 illustrates significant differences in decoding accuracy across frequency bands (Kruskal-Wallis test, p < 0.001), with the theta band showing the highest accuracy (79.47%) in distinguishing between temporal and identity information. Next, we performed Wilcoxon signed-rank tests on the mean decoding accuracies within each band across subjects. Frequency bands with decoding accuracies exceeding the average value were selected for further analysis of their graph metrics. These included the theta, low-gamma, and high-gamma bands.

**Fig 6:**
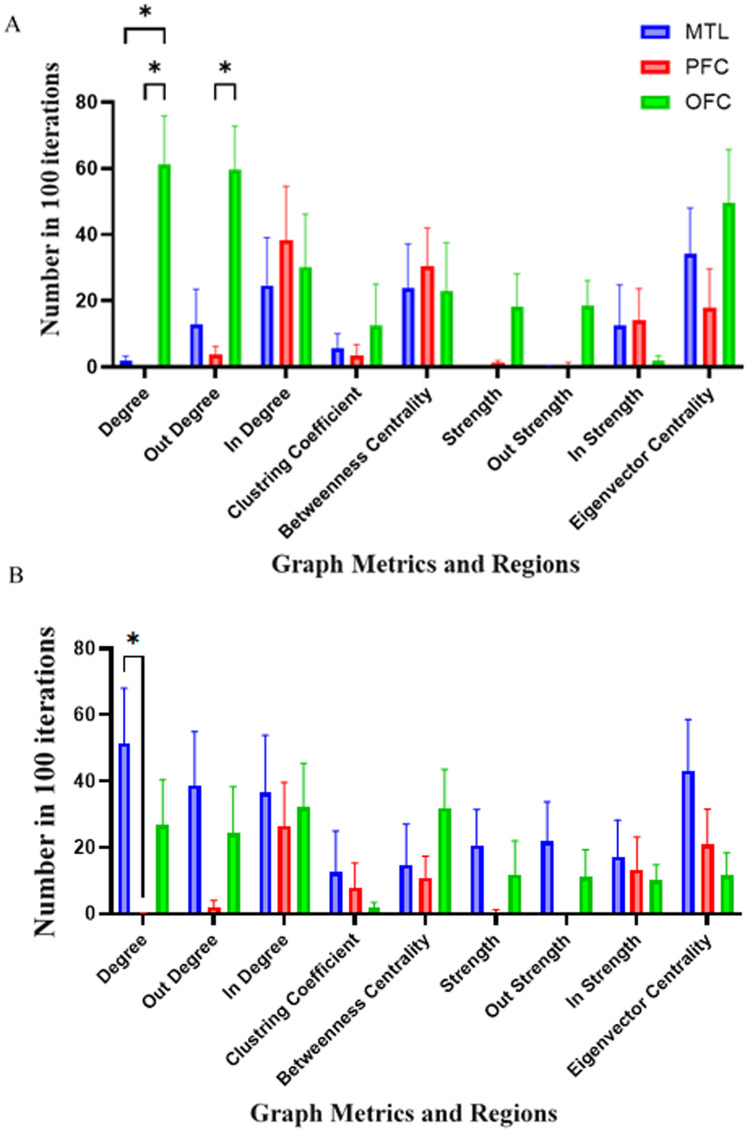
Feature importance in Temporal-Identity condition in the theta band. A two-way repeated measures ANOVA was conducted to examine the interaction between Regions (MTL, PFC, OFC) and Features in the context of degree and out-degree metrics. The horizontal axis shows different graph metrics and the vertical axis shows the mean of scores among 100 iterations. A) The analysis in the theta revealed a significant interaction in regions (p = 0.017, F (1.66, 11.6) = 6.34). Post-hoc Tukey’s multiple comparisons tests identified that, in the degree metric, a significant difference was observed between the MTL and OFC (p = 0.014, Mean Difference = 59.5) and between the PFC and OFC (p = 0.01, Mean Difference = 61.3). Additionally, in the out-degree metric, a significant difference was found between the PFC and OFC (p = 0.015, Mean Difference = 55.5). B) Analysis on the low-gamma band revealed Significant interactions in regions within the degree, with differences between MTL and PFC (p = 0.042, Mean Difference = 51.3). Abbreviations: MTL, medial temporal lobe; PFC, prefrontal cortex; OFC, orbitofrontal cortex.

In the subsequent graph-based analyses, significant effects were observed in the theta and low-gamma bands, while no significant differences were found in the high-gamma band. Accordingly, detailed results for the theta and low-gamma bands are presented in the main text (Fig 6A and Fig 6B), whereas the results for the high-gamma band are provided in the Supplementary Materials (S2 Fig) for completeness and transparency. According to Fig 6A, in the theta band, interaction effects and regional differences in graph metrics by a two-way repeated measures ANOVA was analyzed. Analysis revealed a significant interaction in regions with a p-value of 0.017, F (1.66, 11.6) = 6.34. Post-hoc Tukey’s multiple comparisons tests identified that, in the degree metric, a significant difference was observed between the MTL and OFC (p = 0.014, Mean Difference = 59.5) and between the PFC and OFC (p = 0.01, Mean Difference = 61.3). In the out-degree metric, a significant difference was found between the PFC and OFC (p = 0.015, Mean Difference = 55.5). No significant differences were detected among graph metrics as features or in the interaction of regions and features. Additionally, according to Fig 6B, in the low-gamma band, a significant difference in the degree metric was identified between the MTL and PFC (p = 0.042, Mean Difference = 51.3). The complete results for the high-gamma band, which did not show significant effects, are presented in Supplementary S2 Fig.

These findings are consistent with our second hypothesis, which anticipated that temporal processing would rely on theta band connectivity, with the MTL and OFC contributing to sequence encoding and temporal control. The observed significant differences in degree and out-degree metrics among the MTL, PFC, and OFC within the theta band support the proposed role of these regions in temporal-identity processing. Additionally, the involvement of the low-gamma band in differentiating MTL and PFC connectivity aligns with the idea that multiple frequency bands contribute to feature-specific processing in WM networks. Collectively, these results validate the hypothesized frequency- and region-specific organization of working memory processes for temporal and identity information.

### Alpha band connectivity highlights regional and graph metric differences in distinguishing temporal and spatial processing

**Fig 7:**
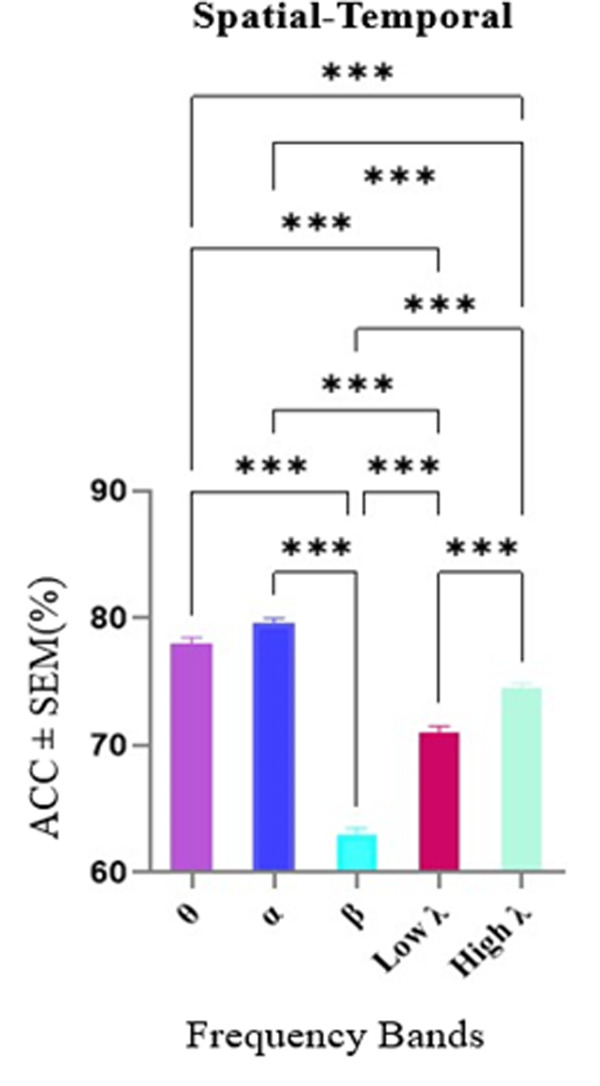
Decoding accuracy in Spatial-Temporal condition across frequency bands. The horizontal axis shows different frequency bands and the vertical axis shows the average accuracies ± SEM. The average decoding accuracies in the alpha frequency band were significantly higher than other bands. Data were analyzed by Kruskal-Wallis test and multiple comparison tests (**) p < 0.01, (***) p < 0.001, (****) p < 0.0001.

Alpha band spectral activity emerged as a critical neural correlate for differentiating between spatial and temporal conditions in WM. As shown in Fig 7, a Kruskal-Wallis test revealed significant differences in decoding accuracy across frequency bands (p < 0.001), with the alpha band showing the highest decoding accuracy (79.60%) compared to all other bands, except for theta where no significant difference was observe. In contrast, the beta band exhibited significantly lower accuracy compared to the other bands.

Following this, we examined graph metrics in frequency bands whose decoding accuracies exceeded the average value across bands. These included alpha, theta, low-gamma, and high-gamma bands. However, subsequent graph metric analyses indicated that only the alpha band yielded statistically significant effects in this context. The detailed outcomes for theta, low-gamma, and high-gamma bands, which did not reach significance, are provided in the supplementary (S3 Fig).

**Fig 8:**
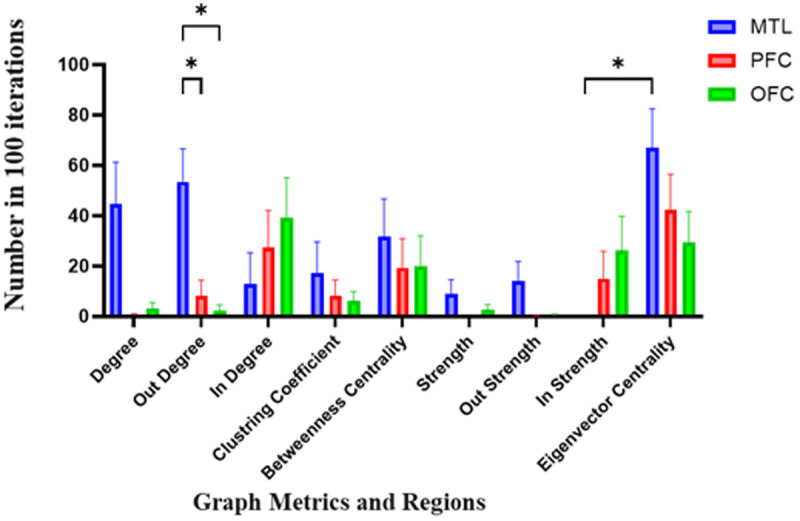
Feature importance in Spatial-Temporal condition in the alpha band. Interaction effects between Regions and Graph Metrics in the theta band was conducted to by a two-way repeated measures ANOVA. The horizontal axis shows different graph metrics and the vertical axis shows the mean of scores among 100 iterations. A significant interaction was observed with a p-value of 0.046 and F (3.75, 26.2) = 2.86. Post-hoc Tukey’s multiple comparisons tests revealed significant differences in the Out-Degree Metric between the MTL and PFC (p = 0.034, Mean Difference = 45.4) and the MTL and OFC (p = 0.023, Mean Difference = 51.1). Additionally, within the MTL, the In-Strength Metric was significantly different from eigenvector centrality (p = 0.044, Mean Difference = −67.1). These findings underscore the distinct role of graph metrics in differentiating regional connectivity patterns. Abbreviations: MTL, medial temporal lobe; PFC, prefrontal cortex; OFC, orbitofrontal cortex.

Fig 8 illustrates the significance of different graph metrics within the alpha band with a two-way repeated measures ANOVA. The analysis revealed a significant interaction between Regions and Graph Metrics, with a p-value of 0.046 and F (3.75, 26.2) = 2.86. Post-hoc Tukey’s multiple comparisons tests provided further insights. In the Out-Degree Metric, significant differences were observed between the MTL and PFC (p = 0.034, Mean Difference = 45.4) and between the MTL and OFC (p = 0.023, Mean Difference = 51.1). Within the MTL, a significant difference was identified in the In-Strength Metric when compared to eigenvector centrality (p = 0.044, Mean Difference = −67.1).

These findings align with our third hypothesis, which proposed that spatial-temporal processing would be characterized by higher classification accuracy in the alpha band, with the MTL exhibiting distinct patterns of outward information flow, reflecting its integrative role in combining spatial and temporal information. The observed significant differences in out-degree and in-strength metrics, particularly within the MTL, confirm its central involvement in coordinating spatial and temporal information during WM tasks. These results provide clear evidence for frequency- and region-specific network dynamics supporting complex WM processes, thereby supporting our theoretical predictions.

### Neural correlates predict response times in WM

The mean ± SEM of RT for correct trials were as follows: identity condition: 1659 ± 701 ms, spatial condition: 1335 ± 574 ms, and temporal condition: 1475 ± 734 ms. Statistical analysis revealed a significant difference in RT between identity-spatial and identity-temporal trials (Kruskal-Wallis, p < 0.001).). Post-hoc pairwise comparisons were conducted using Dunn’s test, which confirmed significant differences between identity-spatial (p < 0.001) and identity-temporal (p < 0.001) conditions. However, there was no significant difference in RT between temporal-spatial trials (p = 0.729).

**Fig 9:**
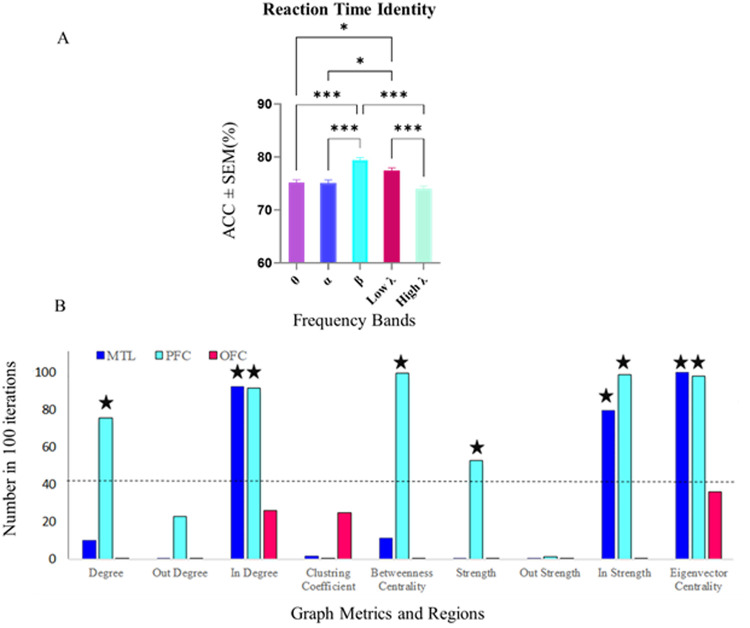
Decoding accuracy and significant feature selection for fast and slow RT classification in Identity trials within the beta band. A) Decoding accuracy for fast and slow RT across frequency bands in Identity trials. A significant increase in decoding accuracy was observed in the beta band (79.21%) compared to theta, alpha, and high-gamma bands (Kruskal-Wallis, p < 0.001), emphasizing the critical role of beta band activity in distinguishing reaction times during identity-based working memory tasks. Data were analyzed by Wilcoxon-test and multiple comparison tests: (*) p < 0.05, (**) p < 0.001, (****) p < 0.0001. B) Significant feature selection frequency across regions and graph metrics within the beta band. Features exceeding the binomial distribution threshold of chance level (43 selections out of 100 iterations, p = 0.05) are marked. In the PFC, significant graph metrics included degree, in-degree, strength, in-strength, betweenness centrality, and eigenvector centrality. In the MTL, significant features included in-degree, strength, in-strength, and eigenvector centrality. No features in the OFC exceeded the significance threshold. These results highlight the critical contributions of the PFC and MTL in distinguishing fast and slow RTs in Identity trials. Abbreviations: PFC, prefrontal cortex; MTL, medial temporal lobe; OFC, orbitofrontal cortex.

To explore the distinctions between fast and slow RTs and the role of graph metrics and frequency bands in decoding them, we employed the same methodologies on the Spatial, Identity, and Temporal conditions separately, where each type of trial was concatenated (i.e., Identity trials were put together). Notably, in the Identity condition, Fig 9A, illustrates that a Kruskal-Wallis test revealed significant differences in decoding accuracy across frequency bands (p < 0.001). Decoding accuracy in the beta band (79.21%) was significantly higher for distinguishing between fast and slow reaction times compared to the theta, alpha, and high-gamma bands. This finding suggests that the beta band plays a crucial role in differentiating between fast and slow RT specifically in the Identity trials. The increased accuracy in decoding these RT further highlights the potential significance of the beta band in capturing and distinguishing cognitive processes related to identity-based WM. Additionally, the low-gamma band also demonstrated a decoding accuracy above the average of all frequency bands (p < 0.05). The corresponding graph is provided in the Supplementary.

To identify significant features contributing to the classification of fast and slow RT in the Identity trials within the beta band, we analyzed the frequency of feature selection across regions and graph metrics. Features were considered significant if their selection frequency exceeded the critical value of 43, determined based on the binomial distribution threshold for chance level (p = 0.05). The analysis revealed several graph metrics in specific regions consistently selected above chance (Fig 9B). In the PFC significant features included degree, in-degree, strength, in-strength, betweenness centrality, and eigenvector centrality, underscoring the PFC’s crucial role in distinguishing fast and slow trials. The MTL exhibited significant features, including in-degree, in-strength, and eigenvector centrality, indicating its substantial yet focused contribution to task-related connectivity patterns. In contrast, no graph metrics in the OFC exceeded the significance threshold, highlighting the OFC’s limited involvement in this classification task within the beta band.

Additionally, in the low-gamma band, several graph metrics surpassed the significance threshold (S4 Fig, Supplementary). In the MTL, degree and eigenvector centrality were consistently selected above chance, suggesting this region’s role in modulating fast and slow RT in the low-gamma range. In the PFC, multiple graph metrics, including out-degree, degree, clustering coefficient, betweenness centrality, and in-strength, exceeded the critical value, indicating a diverse and active contribution of the PFC in this band. Furthermore, in the OFC, both out-degree and eigenvector centrality were identified as significant features, pointing to a selective yet meaningful involvement of the OFC in fast and slow RT classification within the low-gamma band.

The findings highlight distinct contributions of the PFC and MTL in predicting reaction times and managing task demands during WM tasks. The PFC showed broad involvement across multiple graph metrics, reflecting its central role in cognitive control, while the MTL demonstrated focused engagement in memory-related processes. The OFC showed limited involvement in RT differentiation. Additionally, while the beta band exhibited the highest decoding accuracy in distinguishing fast and slow RTs in Identity trials, the low-gamma band also contributed meaningfully, with significant graph metrics observed in the PFC, MTL, and selectively in the OFC. Together, these results emphasize the complementary roles of beta and low-gamma oscillations in supporting identity-based WM performance.

**Fig 10:**
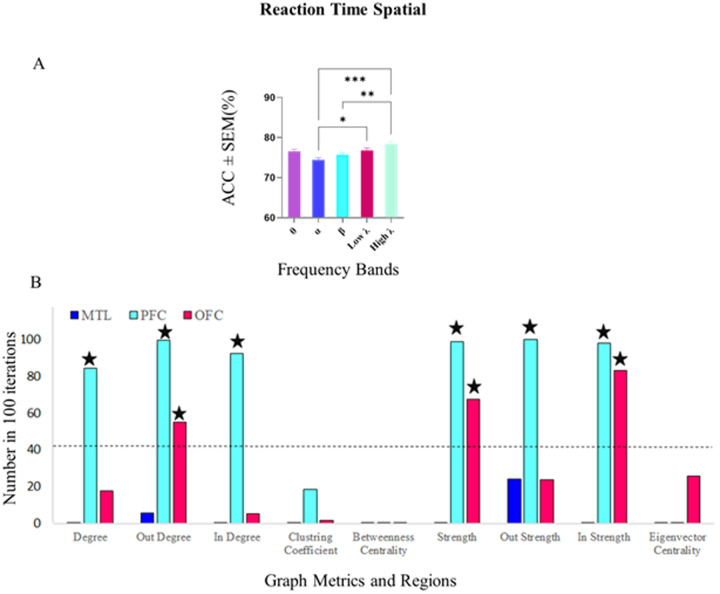
Decoding accuracy and significant feature selection for fast and slow RT classification in Spatial trials within the high gamma band. A) Decoding accuracies for fast and slow reaction times (RTs) during spatial processing across frequency bands. Accuracies in the high-gamma band (78.48%) were significantly higher compared to the alpha and beta bands (Kruskal-Wallis, p < 0.001). No significant differences were observed among the other frequency bands (Kruskal-Wallis, p > 0.05). Data were analyzed using the Kruskal-Wallis test and multiple comparison tests (*p < 0.05, ***p < 0.001, ****p < 0.0001). B) Feature selection patterns in the high-gamma band during Spatial trials. Significant features in the Prefrontal Cortex (PFC) included degree, out-degree, in-degree, strength, out-strength, and in-strength, emphasizing the PFC’s central role in distinguishing fast and slow RTs. The Orbitofrontal Cortex (OFC) exhibited significant features such as out-degree, strength, and in-strength, indicating a more targeted contribution to task-related connectivity. The Medial Temporal Lobe (MTL) showed no significant features, highlighting its minimal involvement in this classification task. Regional contributions reflect distinct roles in spatial processing, with the PFC playing a dominant role and the OFC providing secondary yet notable engagement.

In terms of fast-slow decoding during spatial processing, a Kruskal-Wallis test indicated significant differences in decoding accuracy across frequency bands (p < 0.001). Specifically, decoding accuracy in the high-gamma band (78.48%) was significantly higher than in the alpha and beta bands. No significant differences were observed among the remaining frequency bands (p > 0.05), as it shown in Fig 10A. Moreover, both the alpha and high-gamma bands demonstrated decoding accuracies above the mean accuracy of all subjects (p < 0.05), indicating their notable involvement in spatial RT classification.

The feature selection analysis revealed distinct patterns of involvement across regions within the high gamma band during Spatial conditions. According to Fig 10B, in the PFC, significant features included degree, out-degree, in-degree, strength, out-strength, and in-strength, underscoring the PFC’s central role in distinguishing fast and slow RTs. In the OFC, significant features were out-degree, strength, and in-strength, reflecting a meaningful and more targeted contribution of this region to task-related connectivity patterns. In contrast, no graph metrics exceeded the significance threshold in the MTL, highlighting its minimal involvement in this classification task.

In the alpha band (S5 Fig, Supplementary), significant features in the MTL included degree, out-degree, and out-strength. In the PFC, several graph metrics; degree, out-degree, clustering coefficient, strength, out-strength, and in-strength were selected above chance, highlighting this region’s diverse engagement in fast-slow RT differentiation during spatial processing.

These findings underline the differential roles of brain regions across frequency bands, with the PFC showing consistent involvement, and the alpha and high-gamma bands emerging as key contributors to RT decoding during spatial working memory tasks.

**Fig 11:**
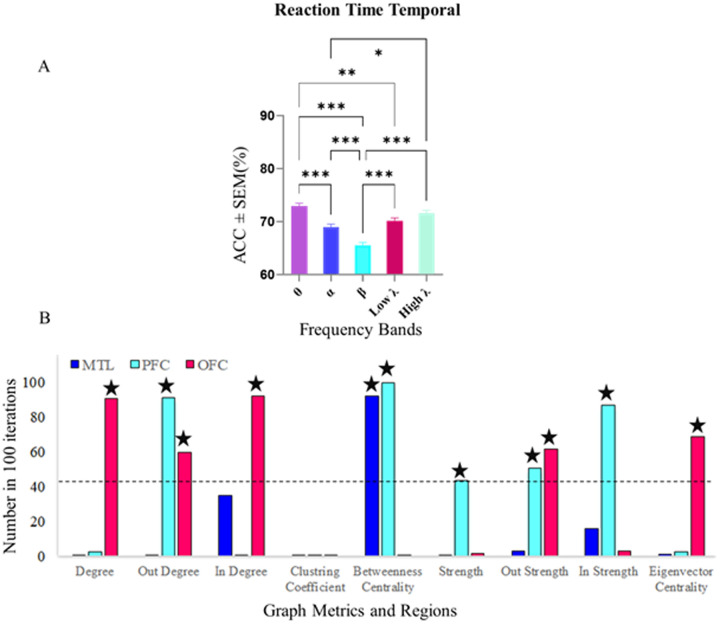
Decoding accuracy and significant feature selection for fast and slow RT classification in Temporal trials within the Theta band. A) Fast-slow decoding accuracies during Temporal processing across frequency bands. The theta band demonstrated the highest decoding accuracy at 72.88%, significantly surpassing the alpha, beta (Kruskal-Wallis, p < 0.001), and low-gamma bands (Kruskal-Wallis, p < 0.01). In contrast, the beta band exhibited the lowest accuracy at 65.51%, highlighting notable differences in decoding performance among the bands. Data were analyzed by Wilcoxon-test and multiple comparison tests (*p < 0.05, **p < 0.01, **p < 0.001). B) Significant graph metrics contributing to fast-slow RT classification within the theta band during Temporal trials. The Prefrontal Cortex (PFC) exhibited prominent involvement with metrics such as out-degree, strength, out-strength, and betweenness centrality surpassing chance levels. The Orbitofrontal Cortex (OFC) showed significant contributions with features including degree, out-degree, in-degree, out-strength, in-strength, and eigenvector centrality. The Medial Temporal Lobe (MTL) displayed selective engagement, with only betweenness centrality exceeding the significance threshold. These findings emphasize the PFC’s central role, the OFC’s integral contributions, and the MTL’s specialized involvement in decoding temporal processing.

Regarding fast-slow decoding during Temporal processing, the Fig 11A illustrates that a Kruskal-Wallis test revealed significant differences in decoding accuracy across frequency bands. Decoding accuracy in the theta band (72.88%) was significantly higher than in the alpha and beta bands (p < 0.001), as well as the low-gamma band (p < 0.01). In contrast, the beta band exhibited the lowest decoding accuracy (65.51%) compared to the other frequency bands.

The analysis identified significant graph metrics that distinguished fast and slow RTs within the theta band during the Temporal condition in Fig 11B. The PFC demonstrated a central role with metrics such as out-degree, strength, out-strength, and betweenness centrality consistently selected above chance levels. The OFC also contributed significantly, with features including degree, out-degree, in-degree, out-strength, in-strength, and eigenvector centrality indicating its integral role in task-related connectivity patterns. The MTL, in contrast, showed limited engagement, with only betweenness centrality which passing the significance threshold, highlighting its more focused involvement.

These findings emphasize the differential roles of brain regions and connectivity metrics in decoding temporal processing within WM tasks. The theta band emerged as the dominant frequency band for decoding fast and slow RTs, consistent with its established involvement in temporal organization and cognitive processing. The PFC takes on a central role in modulating task performance, supported by a diverse array of connectivity metrics, while the OFC contributes integrative and evaluative processing. In contrast, the MTL provides a more targeted but essential contribution, mediating temporal information flow. Collectively, the results from this study clearly validate the proposed hypotheses and provide a more detailed mapping of the brain’s frequency and network interactions in different WM processes.

## Discussion and conclusion

### 1. Introduction and overall findings

The human brain exhibits specialized processing for different types of information within WM [74]. Our study investigated the neural basis of WM’s processing of identity, spatial, and temporal information by investigating brain connectivity [75]. The task involved presenting subjects with two common shapes in specific temporal and spatial locations, followed by a delay period during which WM processed spatial or temporal information while maintaining the identity information of the shapes, or simply remembering the identity of the shapes [10,12,24,25,38,76]. Dynamic interactions recorded from intracranial patients performing the same task revealed that the processing of spatiotemporal information, which was more reciprocal within the delta-theta range compared to the processing of identity. The alpha-beta connectivity did not show any sensitivity to the contents of the WM [25]. Another study, which aimed to separate WM’s ability to recall the location and timing of shapes and examined interelectrode phase synchrony and directional connections, revealed that task-related interactions between the frontal and temporal regions in the theta frequency band were revealed [24]. Patients with PFC damage exhibited impaired WM for spatiotemporal information compared to healthy controls. While the PFC and posterior brain regions interact in delta-theta patterns for spatiotemporal processing, the posterior cortex can support some WM functions independently of the PFC [76]. In another study using the same task, graph theory analysis revealed parieto-occipital alpha-gamma coupling coordinating the selection of spatial and temporal information in control subjects. The signature of feature selection was disturbed in patients with PFC lesions [12]. Another study using this task showed that lesions in the OFC specifically diminished WM’s capacity to retain temporal sequences, while leaving spatial position memory intact [75]. Our study employed graph metrics to analyze effective connectivity within brain networks. Overall, our analysis revealed distinct patterns of classification accuracy across different frequency bands for processing spatial, identity, and temporal information, as well as reaction time. Lower frequency bands, particularly the theta band, demonstrated significant differentiation in spatial-identity and temporal-identity processing. In the spatial-identity condition, the PFC played a key role in distinguishing graph metrics, whereas in temporal-identity conditions, significant interactions were observed between the MTL, PFC, and OFC, with the OFC emerging as a critical region for temporal processing. Additionally, in spatial-temporal processing, the alpha band showed higher accuracy, with the MTL showing a prominent role characterized by distinct patterns of outward information flow, suggesting its contribution to the integration of spatial and temporal information. These findings provide a comprehensive understanding of the neural mechanisms involved in WM and shed light on the functional significance of different brain regions and frequency bands in processing specific types of information.

To further interpret the network dynamics, we selectively compared specific graph metrics and brain regions based on their statistical significance in the post-hoc analyses following the two-way repeated measures ANOVA. This approach allowed us to focus on those pairwise comparisons where meaningful differences emerged, thereby providing clearer insight into (1) how connectivity patterns varied across brain regions within each frequency band, and (2) which graph metrics contributed more prominently to decoding performance. By reporting only the statistically significant comparisons in the main figures, and including the full set of comparisons in the supplementary, we aimed to balance interpretability and completeness while emphasizing the most informative network features related to task demands.

Our results largely supported the hypotheses. In line with the first hypothesis, graph metrics in the theta band revealed significant differentiation in spatial-identity conditions, with the PFC demonstrating a key role in distinguishing connectivity patterns. Supporting the second hypothesis, effective connectivity analyses identified significant interactions between the MTL, PFC, and OFC in temporal-identity processing, with the OFC emerging as a critical node within the theta frequency band. Consistent with the third hypothesis, spatial-temporal processing was characterized by higher classification accuracy in the alpha band, with the MTL exhibiting distinct patterns of outward information flow, suggesting its integrative role in combining spatial and temporal information. Finally, in agreement with the fourth hypothesis, beta band activity within the PFC was associated with reaction time decoding, as indicated by significant graph metrics differentiating faster versus slower reaction time trials.

These findings validate our initial assumptions about the frequency-specific and region-specific organization of WM networks, and provide further evidence for the specialized and dynamic nature of large-scale brain connectivity underlying the maintenance and manipulation of identity, spatial, and temporal information.

In the next subsections, we will delve into the specific findings and implications of spatial processing, identity processing, temporal processing, and reaction time decoding.

### 2. Neural correlates of spatial processing

Spatial processing is a fundamental aspect of WM. In this subsection, we discuss the results related to spatial processing and its neural correlates, shedding light on the specific mechanisms involved in representing and discriminating spatial information within WM.

By comparing the classification accuracy between spatial and identity conditions, we were able to isolate the neural correlates specific to spatial processing. In this task, subjects were instructed to remember specific common shapes in spatial and temporal order. Using a test cue, it was possible to isolate spatial and temporal aspects of WM from the maintenance of item identity [24]. Thus, classifying spatial versus identity trials provided insights into the neural mechanisms underlying spatial processing specifically.

Our findings emphasize the critical role of the PFC and theta band in WM processes during the comparison between spatial and identity conditions. Graph metrics offered deeper insights into the underlying network dynamics, as summarized below: Regarding Strength and Out-Strength, significant differences were observed in the PFC, indicating its substantial contribution to the distribution and integration of information in processing spatial-identity. Strength measures the cumulative weight of connections to and from a node, while out-strength focuses specifically on outgoing connections, reflecting the PFC’s active involvement in distributing information to other brain regions during task execution. For Eigenvector Centrality, differences between strength and eigenvector centrality were observed that suggest although the PFC exhibits strong connectivity (strength), the influence of these connections within the overall network (eigenvector centrality) may vary. This indicates that certain channels within the PFC play a pivotal coordinating role, facilitating effective interactions among other channels and supporting the integration of spatial information across distributed neural networks during WM tasks. Theta band oscillations are widely associated with cognitive processes like WM and attention. The observed high decoding accuracy and interaction effects in this band suggest that PFC activity during spatial-identity tasks is synchronized and critical for maintaining and manipulating WM content. These findings align with our hypothesis that mentioned the role of the PFC in maintaining spatial information in WM [13–16], and also support prior findings indicating connectivity in theta was heightened in spatiotemporal WM [77–79].

### 3. Neural correlates of temporal processing

Temporal processing is another important aspect of WM, involving the encoding and maintenance of temporal information. In this subsection, we discuss the results related to temporal processing and its neural correlates, shedding light on the specific mechanisms involved in representing and discriminating temporal information within WM. Our analysis focused on the classification between identity and temporal conditions, allowing us to examine the neural correlates underlying temporal processing. The goal was to investigate the importance of different frequency characteristics, brain regions, and graph criteria in distinguishing between these two types of information.

The findings highlight the wide range of contributions of the theta band and the OFC in temporal and identity processing. The functional role of specific graph metrics is as follows: Degree measures the number of direct connections to a node, representing its centrality in the network. The observed significant differences in degree between the OFC and both the PFC and MTL suggest the OFC’s role as a key to unite temporal and identity information. Out-Degree focuses on outgoing connections. Our findings showed the OFC’s active role in spreading temporal and identity information in processing and transmitting information to other regions, particularly the MTL. This aligns with the known involvement of the MTL, particularly the hippocampus, in temporal encoding and episodic memory retrieval [80,81]. Theta oscillations were considered a fundamental base of temporal sequencing, memory formation, and the integration of information across time. High decoding accuracy in the theta band suggests that temporal and identity processing rely on coordinated activity in this frequency range, particularly within the OFC. This finding is consistent with previous evidence highlighting the critical role of theta oscillations in the maintenance of temporal information in WM [82]. Furthermore, the significant differences between regions in the degree and out-degree metrics highlight the OFC’s dominance in these conditions.

In addition to the theta band findings, the low-gamma band also demonstrated a notable contribution in differentiating brain regions. Specifically, a significant difference in the degree metric was identified between the MTL and PFC, indicating distinct patterns of connectivity and information integration between these regions during temporal and identity processing. This finding suggests that while theta oscillations predominantly support large-scale temporal organization, low-gamma connectivity may facilitate more localized, high-frequency interactions essential for information exchange between key memory-related structures such as the MTL and PFC. These results complement previous evidence emphasizing the role of gamma-band activity in memory retrieval and working memory maintenance, particularly through dynamic coupling between prefrontal and medial temporal structures.

These results indicate that frequency-specific connectivity, especially in the theta and low-gamma bands, alongside regional network dynamics, play key roles in representing temporal and identity information in WM, with the OFC and PFC showing prominent involvement. These results are aligned with findings related to encoding and processing spatiotemporal information in the MTL [24,25,83]. Moreover, it has been seen that theta frequency bands in EEG carry content of WM and different cognitive processes [25,84].

### 4. Temporal vs spatial classification analysis

The classification analysis comparing temporal and spatial trials allowed us to investigate the differences in neural activity and processing mechanisms associated with these two types of information within WM.

Alpha band activity found to be fundamental for the spatial-temporal decoding [24]. As in a study the link between alpha activity and spatial attention was highlighted, moreover, there are a powerful approach for monitoring spatial and temporal dynamics of spatial information [85,86]. Moreover, the channels in the MTL and PFC demonstrated greater influence in the spatial-temporal decoding due to their highly selected eigenvector centrality values. The outflow of information from the MTL, as reflected by significantly higher out-degree values, was markedly greater than that from the OFC and PFC. Therefore, the information flow entering the channels of the PFC and OFC showed distinct patterns, likely attributable to the differential in-degree values in these two areas. Thus, various brain regions contribute to the differentiation of temporal and spatial information during WM tasks.

The findings underscore the importance of alpha band connectivity and specific graph metrics in distinguishing between spatial and temporal processing. Out-degree represents outgoing flow of information, which reflects the spreading of information from one region to others. The significant differences in out-degree between the MTL and PFC/OFC demonstrate how different brain regions play different but complementary roles in transmitting information, and how information is propagated across brain networks is important for supporting different cognitive tasks such as memory, decision-making, and attentional control. The PFC and OFC, are lower out-degree mean values compared to the MTL. In-strength measure the cumulative weight of incoming connections to a node. The significant differences between in-strength and eigenvector centrality within the MTL suggest unique dynamics in how the MTL integrates and processes spatial and temporal inputs. The very little value in-strength emphasize the MTL’s role as a minimum role of input information from other brain regions. These results suggest unique patterns of information integration in the MTL, with in-strength indicating minimal direct input from other regions, while higher eigenvector centrality reflects broader network influence. The high decoding accuracy in the alpha band reinforces its importance in differentiating between spatial and temporal WM tasks. The results suggest that the MTL, particularly within the alpha frequency band, plays a role in integrating and relaying information for task-specific demands.

### 5. Reaction time

Behavioral variables are routinely collected in human neuroimaging studies, along with functional and effective data. Therefore, brain connection could predict individual differences in behavior, and it is a considerable goal of modern neuroscience. In this subsection, using connectivity data, we predict behaviors and traits, and investigate the influence of different graph metrics and frequency bands on decoding fast and slow RT during WM tasks. Prediction is a synonym of correlation which it means brain feature ‘x’ can predict a behavioral feature ‘y’, where x is derived from effective connectivity and y represents task performance regarding slow/fast RT [87].

Our analysis revealed that the beta band stood out as the most discriminative, particularly in identity condition. The PFC values exerted a substantial impact on the classification, as indicated by their high selection rates. Notably, a large proportion of selected values were derived from the PFC, suggesting its crucial involvement in modulating reaction speed. It is along with the other studies which discuss the role of the PFC in executive function. 193 neuroimaging studies showed the patterns of activation across the PFC [88]. The PFC neurons represent shifts in behavior [2].

The higher decoding accuracy observed in the beta band for differentiating fast and slow RTs suggests that beta oscillations play a key role in modulating task-related cognitive processes, particularly in identity-based WM. The PFC illustrated a wide use of significant graph metrics, including degree, in-degree, strength, in-strength, betweenness centrality, and eigenvector centrality. These metrics reflect the PFC’s essential role in modulating cognitive flexibility, attention, and decision-making processes, all of which are critical in distinguishing fast from slow RTs. The lack of use of out-degree and out-strength suggest that out flow information in the PFC were not remarkable. The degree and strength metrics, indicating the PFC’s ability for information flow, suggest that the PFC’s involvement is especially important in regulating the attention and memory resources needed for fast response decisions. The MTL illustrated engage in metrics in particular, in-degree and in-strength and eigenvector centrality, indicating the MTL’s role as a key receiver and integrator of information within the memory network. The absence of significant features in the OFC for distinguishing fast and slow RTs in identity conditions showed that while the OFC is often involved in sequencing information in WM, it may play a lesser role in modulating cognitive aspects like RT within identity-based WM tasks. In addition, in the low-gamma band, multiple metrics exceeded significance. The PFC demonstrated diverse engagement through out-degree, degree, clustering coefficient, betweenness centrality, and in-strength, while the MTL contributed via degree and eigenvector centrality. Interestingly, the OFC showed selective involvement through out-degree and eigenvector centrality, indicating a modest yet relevant role in modulating reaction times within this frequency range.

For reaction time in spatial condition, we revealed neurobiological significance and functional implications as follows: higher decoding accuracy observed in both the high-gamma and alpha bands for distinguishing fast and slow RTs suggests that these frequency ranges are critically involved in distinct yet complementary cognitive processes during spatial WM tasks. This reveals that high-gamma oscillations are particularly important for more dynamic, complex processing within spatial WM tasks. The high-gamma band, traditionally linked to attention, perception, and memory consolidation, appears to support more dynamic and complex processing demands. In contrast, the involvement of the alpha band typically associated with attentional control and inhibitory processing indicates its role in modulating task-relevant information and suppressing irrelevant activity to optimize performance.

The PFC displayed a wide range of significant graph metrics in both high-gamma and alpha bands, including degree, out-degree, in-degree, strength, out-strength, and in-strength. The degree and strength metrics reflect the PFC’s involvement in processing information from multiple nodes (electrodes), whereas the out-degree and out-strength metrics show the PFC’s role in directing information flow to other regions to facilitate spatial memory processing. These findings illustrated the central role of the PFC in managing cognitive tasks that demand attention and executive function [4,13–15]. The OFC showed significant metrics, including out-degree, strength, and in-strength, suggesting that this region plays a more specialized role in processing spatial WM tasks. The selective engagement of the OFC in these specific metrics suggests that the OFC is involved in spatial memory tasks, especially when responding fast or slow to spatial cues. The Notably, the MTL while not showing significant features in the high-gamma band, exhibited meaningful involvement in the alpha band, where degree, out-degree, and out-strength metrics surpassed the chance level. This suggests a frequency-specific engagement of the MTL in modulating memory-related processes within the alpha range, possibly through selective gating or coordination of memory retrieval in decoding RT during spatial WM tasks.

Regarding reaction time in temporal trials, the findings underscore the critical role of theta band oscillations in distinguishing fast and slow RTs during temporal processing. The theta band is well-established as a neural correlate of memory and temporal organization, and its dominance in decoding accuracy suggests its centrality to the neural mechanisms underlying temporal WM. The PFC’s wide range of significant metrics shows its critical role for processing temporal information. The out-degree and strength metrics reflect its role in both receiving and transmitting information to coordinate task performance, while betweenness centrality highlights its importance in facilitating efficient communication across the neural network. The OFC’s broader engagement in various graph metrics reflects its significant contribution to connectivity patterns essential for temporal processing. Its involvement likely pertains to the evaluative and integrative aspects of temporal tasks, complementing the executive functions performed by the PFC. The MTL’s engagement, though limited, highlights its focused role in organizing and contextualizing temporal information within the WM framework. Its contribution via betweenness centrality emphasizes its role in mediating information transfer between regions during temporal processing. The PFC and OFC demonstrated a high degree of involvement, indicating that these regions play a pivotal role in regulating the speed of information processing and coordinating interactions between other brain regions in temporal trials. As it is illustrated the PFC has the capacity for the temporal organization of future behavior like WM [89].

### 6. Limitations and future directions

This research aims to establish a connection between connectivity patterns and behavioral outcomes; however, the overall high memory accuracy (ranging from 0.79 to 0.97) and the low rate of incorrect trials (below 20%) restricted the potential for meaningful statistical comparisons between successful and unsuccessful trials. In light of this limitation, our analysis focused on decoding mechanisms related to spatial and temporal processing rather than classifying trial outcomes. We recognize this as a constraint and recommend that future investigations utilize more demanding tasks to enhance behavioral variability, thereby allowing for a deeper exploration of the relationship between connectivity patterns and trial-by-trial accuracy, which would strengthen the theoretical framework. Another limitation of our study is the restricted number of intracranial recording sites. Future studies could use high-density EEG or combined fMRI-EEG approaches to further explore these network dynamics in healthy individuals

Overall, the study emphasizes the importance of the wide range of frequency band and the frontal lobe in predicting RT during WM tasks. These results deepen our understanding of the neural processes underlying the variability in reaction time and shed light on the functional significance of different brain regions in WM tasks. Importantly, brain regions don’t work separately, instead they cooperate together to carry WM contents.

## Supporting information

S1 Feature importance scores in the Spatial-Identity conditions across low-gamma band. Each feature was assigned a score ranging from 0 to 100, reflecting its relative contribution to the classification process. The horizontal axis represents different graph metrics, while the vertical axis indicates the mean feature importance scores over 100 iterations. No significant differences were detected in regions or graph metrics in bands. Abbreviations: MTL, medial temporal lobe; PFC, prefrontal cortex; OFC, orbitofrontal cortex.
(TIF)

S2 Feature importance scores in the Temporal-Identity conditions across frequency bands. Each feature was assigned a score ranging from 0 to 100, reflecting its relative contribution to the classification process. The horizontal axis represents different graph metrics, while the vertical axis indicates the mean feature importance scores over 100 iterations. Results of a two-way repeated measures ANOVA on low-gamma band decoding data with region (MTL, PFC, OFC) and graph metric as factors. Significant interactions were observed in regions within the degree, with differences between MTL and PFC (p = 0.042, Mean Difference = 51.3). No significant differences were detected in other regions or graph metrics in other bands. Abbreviations: MTL, medial temporal lobe; PFC, prefrontal cortex; OFC, orbitofrontal cortex.
(TIF)

S3 Feature importance scores in the Spatial-Temporal conditions across frequency bands. Each feature was assigned a score ranging from 0 to 100, reflecting its relative contribution to the classification process. The horizontal axis represents different graph metrics, while the vertical axis indicates the mean feature importance scores over 100 iterations. No significant differences were detected in regions or graph metrics in bands. Abbreviations: MTL, medial temporal lobe; PFC, prefrontal cortex; OFC, orbitofrontal cortex.
(TIF)

S4 Significant feature selection frequency across regions and graph metrics within frequency bands that exhibited above-average decoding accuracy for fast and slow RT classification in Identity trials. Features exceeding the binomial distribution threshold of chance level (43 selections out of 100 iterations, p = 0.05) are marked. Abbreviations: PFC, prefrontal cortex; MTL, medial temporal lobe; OFC, orbitofrontal cortex.
(TIF)

S5 Significant feature selection frequency across regions and graph metrics within frequency bands that exhibited above-average decoding accuracy for fast and slow RT classification in Spatial trials. Features exceeding the binomial distribution threshold of chance level (43 selections out of 100 iterations, p = 0.05) are marked. Abbreviations: PFC, prefrontal cortex; MTL, medial temporal lobe; OFC, orbitofrontal cortex.
(TIF)
