# Supplementary figures and images for: Distinct neural patterns for various information in working memory: A brain connectivity study
Source: PLoS One. 2025 Jul 3;20(7):e0326449. doi: 10.1371/journal.pone.0326449 (PMC12225848; doi:10.1371/journal.pone.0326449)

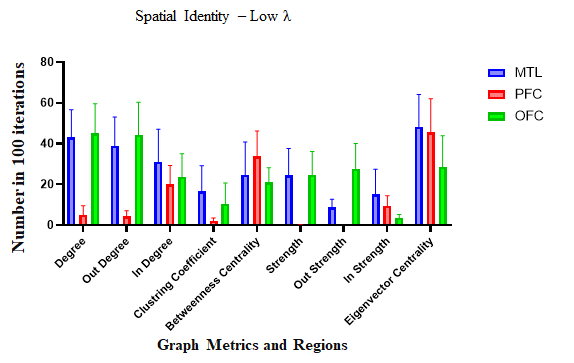

Supplement: S1 — Each feature was assigned a score ranging from 0 to 100, reflecting its relative contribution to the classification process. The horizontal axis represents different graph metrics, while the vertical axis indicates the mean feature importance scores over 100 iterations. No significant differences were detected in regions or graph metrics in bands. Abbreviations: MTL, medial temporal lobe; PFC, prefrontal cortex; OFC, orbitofrontal cortex. (TIF) [file pone.0326449.s001.tif]

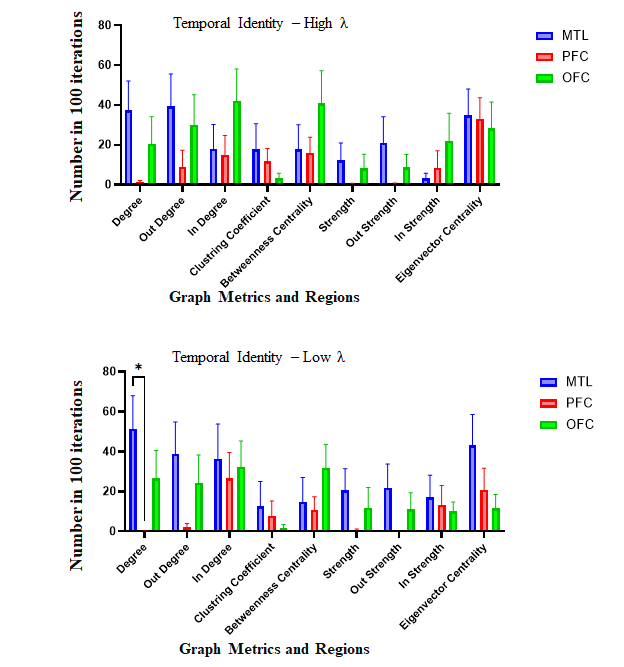

Supplement: S2 — Each feature was assigned a score ranging from 0 to 100, reflecting its relative contribution to the classification process. The horizontal axis represents different graph metrics, while the vertical axis indicates the mean feature importance scores over 100 iterations. Results of a two-way repeated measures ANOVA on low-gamma band decoding data with region (MTL, PFC, OFC) and graph metric as factors. Significant interactions were observed in regions within the degree, with differences between MTL and PFC (p = 0.042, Mean Difference = 51.3). No significant differences were detected in other regions or graph metrics in other bands. Abbreviations: MTL, medial temporal lobe; PFC, prefrontal cortex; OFC, orbitofrontal cortex. (TIF) [file pone.0326449.s002.tif]

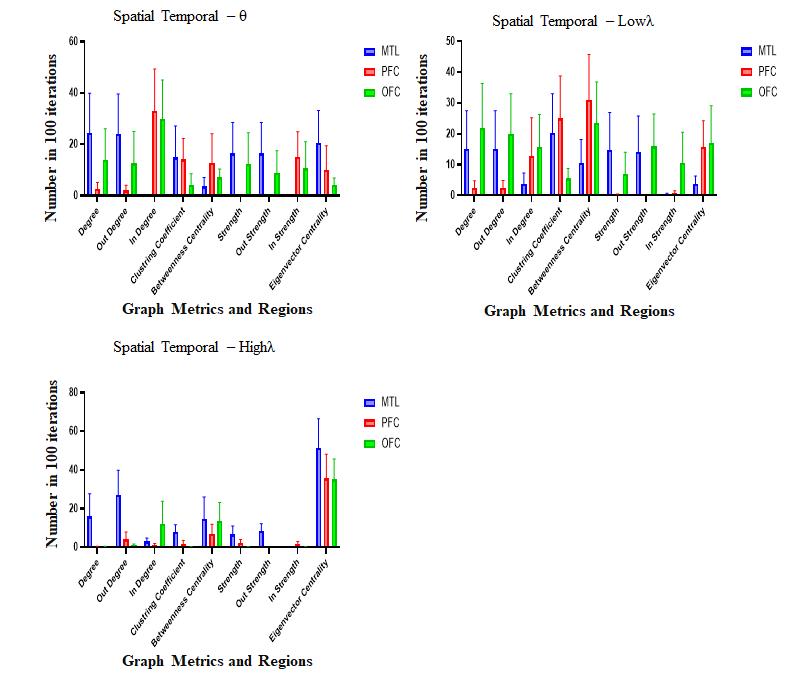

Supplement: S3 — Each feature was assigned a score ranging from 0 to 100, reflecting its relative contribution to the classification process. The horizontal axis represents different graph metrics, while the vertical axis indicates the mean feature importance scores over 100 iterations. No significant differences were detected in regions or graph metrics in bands. Abbreviations: MTL, medial temporal lobe; PFC, prefrontal cortex; OFC, orbitofrontal cortex. (TIF) [file pone.0326449.s003.tif]

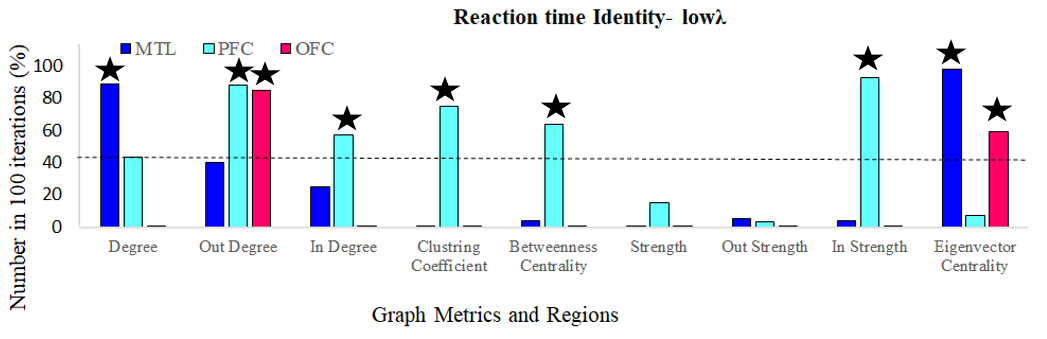

Supplement: S4 — Features exceeding the binomial distribution threshold of chance level (43 selections out of 100 iterations, p = 0.05) are marked. Abbreviations: PFC, prefrontal cortex; MTL, medial temporal lobe; OFC, orbitofrontal cortex. (TIF) [file pone.0326449.s004.tif]

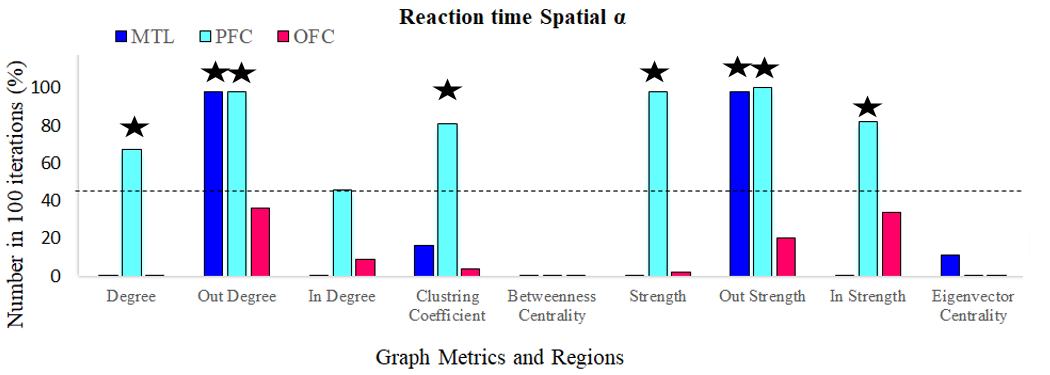

Supplement: S5 — Features exceeding the binomial distribution threshold of chance level (43 selections out of 100 iterations, p = 0.05) are marked. Abbreviations: PFC, prefrontal cortex; MTL, medial temporal lobe; OFC, orbitofrontal cortex. (TIF) [file pone.0326449.s005.tif]
